# *Proteus mirabilis* Targets Atherosclerosis Plaques in Human Coronary Arteries *via* DC-SIGN (CD209)

**DOI:** 10.3389/fimmu.2020.579010

**Published:** 2021-01-08

**Authors:** Ying Xue, Qiao Li, Chae Gyu Park, John D. Klena, Andrey P. Anisimov, Ziyong Sun, Xiang Wei, Tie Chen

**Affiliations:** ^1^ Department of Clinical Immunology, Tongji Hospital, Tongji Medical College, Huazhong University of Science and Technology, Wuhan, China; ^2^ Laboratory of Immunology, Brain Korea 21 FOUR Project for Medical Science, Institute for Immunology and Immunological Diseases, Severance Biomedical Science Institute, Yonsei University College of Medicine, Seoul, South Korea; ^3^ Division of Global Health Protection, Center for Global Health, U.S. Centers for Disease Control and Prevention, Atlanta, GA, United States; ^4^ Laboratory for Plague Microbiology, State Research Center for Applied Microbiology and Biotechnology, Especially Dangerous Infections Department, Obolensk, Russia; ^5^ Department of Clinical Laboratory, Tongji Hospital, Tongji Medical College, Huazhong University of Sciences and Technology, Wuhan, China; ^6^ Division of Cardiothoracic and Vascular Surgery, Tongji Hospital, Tongji Medical College, Huazhong University of Science and Technology, Wuhan, China

**Keywords:** Atherosclerosis plaques, *Proteus mirabilis*, cluster of differentiation (CD) 209, lipopolysaccharide (LPS) core, macrophage

## Abstract

Bacterial DNAs are constantly detected in atherosclerotic plaques (APs), suggesting that a combination of chronic infection and inflammation may have roles in AP formation. A series of studies suggested that certain Gram-negative bacteria were able to interact with dendritic cell-specific intercellular adhesion molecule-3-grabbing non-integrin [DC-SIGN; cluster of differentiation (CD) 209] or langerin (CD207), thereby resulting in deposition of CD209s at infection sites. We wondered if *Proteus mirabilis* (a member of Proteobacteria family) could interact with APs through CD209/CD207. In this study, we first demonstrated that CD209/CD207 were also receptors for *P. mirabilis* that mediated adherence and phagocytosis by macrophages. *P. mirabilis* interacted with fresh and CD209s/CD207-expressing APs cut from human coronary arteries, rather than in healthy and smooth arteries. These interactions were inhibited by addition of a ligand-mimic oligosaccharide and the coverage of the ligand, as well as by anti-CD209 antibody. Finally, the hearts from an atherosclerotic mouse model contained higher numbers of *P. mirabilis* than that of control mice during infection-challenging. We therefore concluded that the *P. mirabilis* interacts with APs in human coronary arteries *via* CD209s/CD207. It may be possible to slow down the progress of atherosclerosis by blocking the interactions between CD209s/CD207 and certain atherosclerosis-involved bacteria with ligand-mimic oligosaccharides.

## Introduction

Cardiovascular diseases caused by atherosclerosis are leading causes of mortality worldwide (1–3). Plaque formation is a hallmark of atherosclerosis (4) and is characterized by deposition of fat, cholesterol and calcium in arterial intima. Atherosclerosis in humans usually affects the “medium” arteries, human coronary, renal, and carotid arteries (5). In general, it is believed that risk factors such as hyperlipidemia, hypertension, obesity, diabetes mellitus, and smoking can contribute to the formation of atherosclerotic plaques (APs) (6–9). However, the detailed molecular and biological mechanisms of atherosclerosis are not clear.

Studies have suggested that risk factors may add to early atherosclerotic lesions in human coronary arteries, which may result in macrophage recruitment (7, 10). In fact, inflammation and macrophage deposition have been observed in atherosclerosis for decades (11–14). Macrophage-derived foam cells are major players in atherosclerosis pathogenesis (15, 16). Initially, it was thought that infiltrated macrophages could phagocytose lipid to eliminate fat *via* scavenger receptors (17–19), but the infiltrated macrophages in turn elicit an inflammatory response that damages vessel walls (20). Several studies have shown that depletion of macrophages in animals results in dramatic reduction of atherosclerosis progression (21–24). Macrophages play a central part in atherosclerosis development and AP destabilization. Hence, scholars have explored therapeutic alternatives by depletion of macrophages and inducing macrophage death (22, 24, 25). Macrophage depletion also promotes AP stability and induces the regression of established APs (24).

An increasing number of studies have shown that infections (particularly chronic infections) are associated with the development of cardiovascular diseases (7–9, 26–30). Bacteria can infect vessel walls directly, thereby leading to their injury and release of inflammatory factors (31). In some circumstances, these bacteria can induce chronic infection residing within antigen-presenting cells (APCs), such as dendritic cells (DCs) or macrophages (30, 32–34). Also, monocytes in the circulation may function as a “Trojan horse” to deliver bacteria from the circulation to vessel walls and sustain the infection (7).

The presence of bacteria in APs has been demonstrated thanks to advancements in DNA-sequencing methods (35–38). For example, DNA from the Gram-negative bacteria *Porphyromonas gingivalis*, *Chlamydia pneumonia* and *Helicobacter pylori* are often detected in human APs (36). One study indicated that the Proteobacteria phylum occupies 48.3% of bacterial profiles in human APs (38). Canducci and colleagues indicated that coronary APs can be stimulated to produce cross-reacting antibodies against *P. mirabilis* (39). We think that Canducci and coworkers might not recognize then that *P. mirabilis* had been present in the APs that were used to produce anti-*P. mirabilis* antibodies. Nevertheless, their study suggested that *P. mirabilis* in APs was could stimulate inflammation. *P. mirabilis* is a member of the Proteobacteria phylum. It is an opportunistic bacterium that can cause urinary-tract infections, induce stone formation and, in some rare cases, cause bacteremia (40, 41).

**Figure 1:**
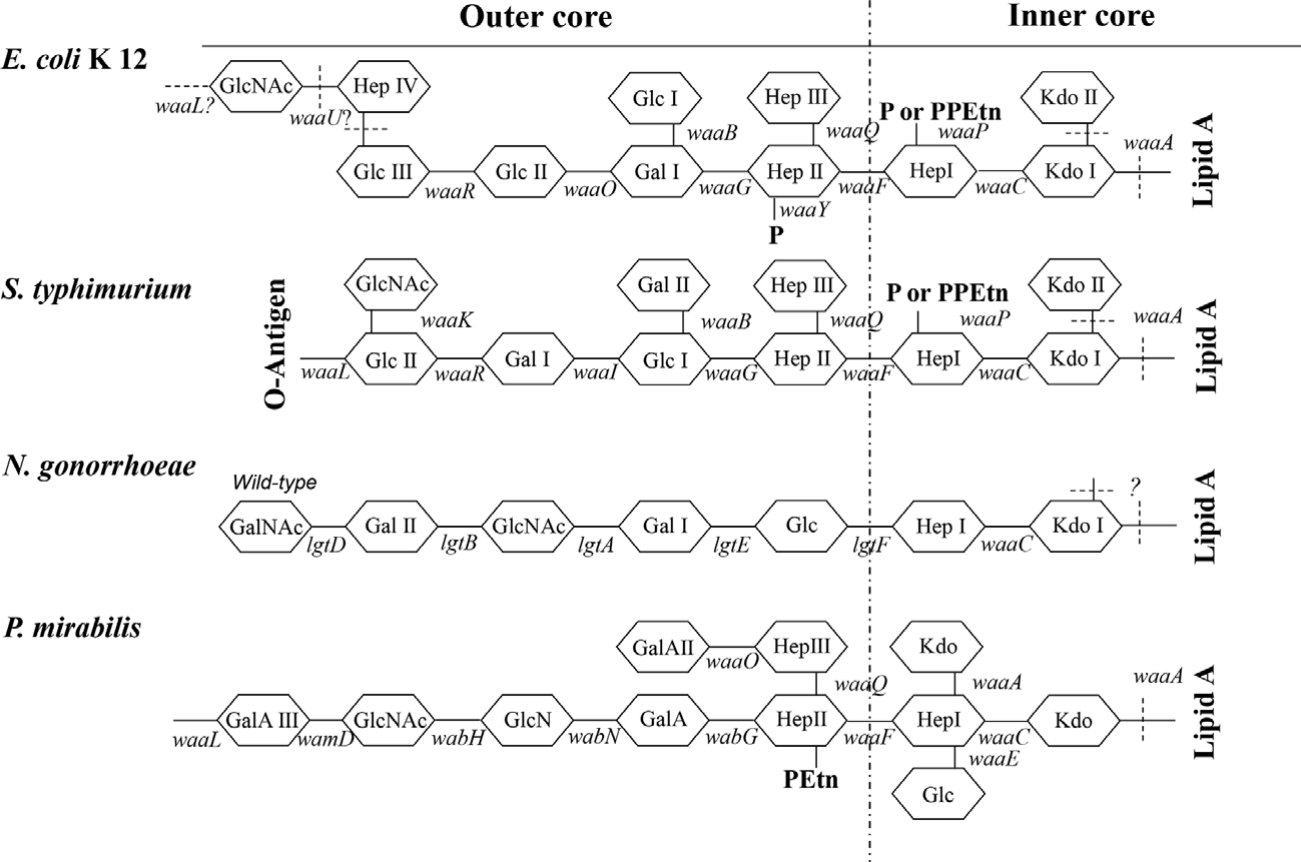
Structure of the LPS core. Structures of the inner-core and outer-core regions of the LPS or LOS of *E. coli* K12, *S. typhimurium*, *N. gonorrhoeae*, and *P. mirabilis* and the genes involved in their synthesis are presented. Genes encoding enzymes that are involved in the biosynthesis of core oligosaccharides are shown along with lines that indicate the approximate sites at which the enzymes function (solid line). Sites that are variably substituted or still under investigation are indicated by dashed lines. GlcNAc, N-acetyl-glucosamine; Glc, glucose; Hep, heptose; Gal, galactose; P, phosphate; PPEtn, phosphoethanolamine; KDO, 2-keto-3-deoxyoctonate. *E. coli* K12 and *N. gonorrhoeae* naturally do not possess the O-antigen.

Structurally, *P. mirabilis* is also a Gram-negative bacterium. Over 70% of the surface of Gram-negative bacteria is occupied by fully expressed lipopolysaccharide (LPS) that, in general, consists of three structural regions (**Figure 1**): (i) lipid A, (ii) the oligosaccharide (LPS) core and (iii) O-polysaccharide (OPS), which is also known as or “O-antigen”. The LPS produced by wild-type Gram-negative bacteria is recognized as “smooth LPS”, whereas LPS lacking the O-antigen is known as “rough LPS” or “lipo-oligosaccharide”. Acting like a shield, O-antigen can promote resistance to serum killing and/or phagocytosis (42). Lipid A is also known as an endotoxin, which are well-documented inducers of inflammation.

A series of studies showed that many Gram-negative bacteria, including Salmonella enterica serovar Typhimurium, Escherichia coli, Yersinia pestis, Yersinia pseudotuberculosis, Neisseria gonorrhoeae, Haemophilus ducreyi, and Shigella spp. can use their LPS core to bind with immune receptors such as dendritic cell-specific intercellular adhesion molecule-3-grabbing non-integrin [DC-SIGN; also known as cluster of differentiation (CD) 209] and human langerin (CD207) (43–51). DC-SIGN and langerin belong to C-type lectins, which are usually expressed by APCs, such as DCs, macrophages, and Langerhans cells (52, 53).

C-type lectins contain one or more carbohydrate-recognition domain that can interact with carbohydrate ligands (54). For example, the carbohydrates on glycoprotein 120 (gp120) of the human immunodeficiency virus (HIV) can bind with DC-SIGN to “hijack” APCs and promote host dissemination of the HIV to T cells in lymph nodes (55, 56). Using similar mechanisms, it has been suggested that Gram-negative *S. typhimurium, Y. pestis* and *Y. pseudotuberculosis* exploit APCs to promote dissemination in the host (47–51). A study by Ye and colleagues on *S. typhimurium*, which causes typhoid fever in mice, speculated that many interactions between CD209 and LPS core could lead to CD209 deposition at infection sites (especially during chronic infections) (51).

DC-SIGN has been detected on APs from the coronary arteries of patients (13). The expressed DC-SIGN on APs was co-localized with the classical macrophage markers CD14 and CD68 (13). Carrione and colleagues found that DC-SIGN-expressing DCs in myeloid cells could facilitate the traffic of *P. gingivalis* (another Gram-negative bacterium) from the oral mucosa to APs (57). Those studies indicated that DC-SIGN on APCs might be involved in atherosclerosis development.

In the present study, we tested the hypothesis that *P. mirabilis* may interact with APs from human coronary arteries through DC-SIGN (CD209).

## Materials and Methods

### Ethics Statement

All animal procedures and human experiments were conducted in strict accordance with the Institutional Animal Care and Use Committees and Institutional Review Board (IRB) of Tongji Hospital, Tongji Medical College, China. The handling of the mice and all experimental procedures were specifically approved for this study by the Medical Ethics Committee of Tongji Hospital and were carried out in accordance with institutional guidelines (TJ-IRB20182677 for animal experiments and TJ-IRB20182315 for human experiments). All procedures on mice were performed under sodium pentobarbital anesthesia, all efforts were made to minimize suffering, and all volunteers involved in the experiment signed informed consent. Twelve plaque samples were retrieved from coronary arteries of patients with fatal coronary heart disease in Tongji Hospital (**Table 1**).

**Table 1:** The clinical information of patients.

Variable	n = 12											
Ages (years)	53	49	41	58	67	44	60	55	60	59	60	58
Gender	Male	Male	Female	Female	Male	Male	Male	Female	Female	Male	Female	Male
Disease diagnosis	Coronary atherosclerotic heart disease											
Type of surgery	Heart transplantation	Coronary artery bypass graft	Coronary artery bypass graft	Coronary artery bypass graft	Coronary artery bypass graft	Coronary artery bypass graft	Coronary artery bypass graft	Heart transplantation	Coronary artery bypass graft	Coronary artery bypass graft	Coronary artery bypass graft	Coronary artery bypass graft

### Biological Reagents

Mannan, the ligand antagonist of the human mannose receptor, was purchased from Sigma (St. Louis, MO, USA). Anti-human DC-SIGN and anti-human Langerin monoclonal antibodies (MAbs) were purchased from Pharmingen (San Diego, CA, USA). Human Glu-plasminogen (Hematologic Technologies, Essex Junction, VT, USA) and the chromogenic plasmin substrate S-2251 (Chromogenix, Milano, Italy) were used in the plasminogen activation assay.

### Bacterial Strains (**Table 2**)

*E. coli* K-12 strain CS180 is *E. coli* with rough LPS missing the O-antigen. CS1861 is the derivative of CS180 transformed with the pSS37 plasmid, which contains all of the necessary genes for O-antigen expression of the *Shigella dysenteriae* 1 (58). CS180-Pla and CS1861-Pla were constructed by CS180 and CS1861 transformed with the pUC18-Pla plasmid expressing the plasminogen activator (PLA) protein from *Y. pestis*, respectively (59).

**Table 2:** Bacterial strains and cell lines used in this study.

Strain or cell line	Characteristics	References
*Y. pseudotuberculosis*		
Y1	O:1a, wild-type expressing invasion but with pYV plamid naturally cured (smooth)	61
		
*Y. pestis*		
1418	Originated from KIM5 (KIM D27) with pgm (pigmentation) gene deleted	60
		
*Y. enterocolitica*	Serotype O:3	This work
*E.coli* K-12		
CS180	Wild-type (rough)	58
CS1861	CS180 expressing O-antigen (smooth)	58
CS180-Pla	CS180 transformed with puc18-Pla plasmid expressing the PLA protein from *Y. pestis*	This work
CS1861-Pla	CS1861 transformed with puc18-Pla plasmid expressing the PLA protein from *Y. pestis*	This work
*P. mirabilis*	Isolated from clinical specimens	This work
*P. mirabilis-*pAY100.1	*P. mirabilis* transformed with pAY100.1 plasmid expressing an O-antigen from *Y. enterocolitica* serotype O:3	This work
*P. mirabilis*-Pla	*P. mirabilis* transformed with puc18-Pla plasmid expressing the PLA protein from *Y. pestis*	This work
P. mirabilis O^+^-Pla	*P. mirabilis* transformed with pss37 and puc18-Pla plasmid expressing an O-antigen from Shigella and the PLA protein from *Y. pestis* respectively	This work
Cell lines		
CHO-NEO cells	Control cell line, which expresses the neomycin resistance gene only	
CHO-hDC-SIGN cells	Generated by transfecting CHO cells with human DC-SIGN cDNAs	
CHO-hLangerin cells	Generated by transfecting CHO cells with human Langerin cDNAs	
Primary macrophage	Primary macrophages from mouse peritoneal cavity	
RAW264.7	Murine macrophage cell line	

*Y. pestis* 1418 originated from KIM5 (KIM D27), a strain, whose *pgm* (pigmentation) gene has been deleted (60). *Y. pseudotuberculosis* Y1 is a serotype O: 1a strain, lacking the virulence plasmid (pYV), was used as a positive control in the cell invasion assay (61).

A *P. mirabilis* strain was isolated from a urine sample from a patient suffering from a chronic and recurrent urinary-tract infection. This patient was recruited from the Department of Clinical Laboratory Medicine of Tongji Hospital (Wuhan, China). *P. mirabilis*-pAY100.1 is *P. mirabilis* that has been transformed by plasmid pAY100.1. *P. mirabilis*-pAY100.1 carries all the genes necessary for expression of the O-antigen of *Yersinia enterocolitica* serotype O:3 (62). *P. mirabilis-*Pla is the strain expressing PLA. *P. mirabilis* O^+^-Pla is *P. mirabilis* that has been transformed with pss37 and pUC-Pla plasmids (59).

### Cell Lines

The CHO-hDC-SIGN and CHO-hLangerin cell lines were generated by transfecting Chinese hamster ovary cells (CHO) cells with corresponding human C-type lectin cDNAs. The transfection was followed by G418 (1.5 mg/ml) selection and screening for stable surface expression. CHO-NEO, which expresses the neomycin resistance gene only, was the control cell line (48–51). The RAW264.7 is a murine macrophage cell line (**Table 2**). All the cells were cultured in DMEM supplemented with 10% of fetal calf serum (FCS), streptomycin (100 μg/ml), and penicillin (100 U/ml) incubated overnight at 37°C with 5% CO_2_.

### Mice

Male C57BL/6J mice, aged 6–8 weeks, were purchased from Hunan SJA Laboratory Animal Co. Ltd (Hunan, China). The mice were housed in animal facilities at the Tongji Hospital in direct accordance with guidelines drafted by the Animal Care Committee of Tongji Hospital.

### Isolation of Mouse Peritoneal Macrophages

Peritoneal macrophages were chosen as our primary cells. After female mice (6 to 8 weeks old) were euthanized, their intact abdomen was exposed, cleaned with 70% ethanol and cut with scissors. A 5 ml aliquot of RPMI was injected into the intraperitoneal cavity. The mouse abdomen was gently massaged for 2 min and then the lavage fluid was collected. The collected macrophages were then used to perform invasion assays. The peritoneal macrophages expressed high levels of SIGNR1 as shown in previous study (46).

### *In Vitro* Cell Invasion Assay with Cell Lines

The cell invasion assays were performed as described previously (63). Host cells (CHO, CHO-hDC-SIGN, CHO-hLangerin, RAW264.7, and peritoneal macrophages) were suspended in RPMI supplemented with 2% FCS. One-half ml of cells was plated in 24-well plates at the concentration of 10^5^cells/well. The RAW264.7 and peritoneal macrophages were seeded onto plates, in which each well contained a 1.5-cm-diameter glass coverside, to allow the cells to adhere quickly. The other host cells were cultured overnight. 10^6^ bacteria in total at the log phage of the growth were used in the cell invasion assay. A 50 µl aliquot of bacterial suspensions was added to the plate at a concentration of 1×10^7^ colony-forming units (CFU)/ml. The cells were incubated with the bacteria at 37°C for 2.5 h in the presence of 5% CO2. The cells were washed three times with PBS. A 2 ml aliquot of 2% FBS RPMI-1640 was added to each well with gentamycin which killed the extracellular bacteria but could not penetrate into the host cells at a final concentration of 100 μg/ml and incubated for 1 h. The cells were washed twice with PBS in the 24-well plate twice. The cells were lysed with 1 ml 1% Triton X-100. The lysate was diluted and put on a LB agar plate. The bacterial colonies recovered from the lysed cells were counted the following day to define the level of internalized bacteria in these host cells. All experiments were performed in triplicate and data are expressed as mean ± standard error.

In the inhibition experiment, the anti-hDC-SIGN/hLangerin antibody was used at a concentration of 5 μg/ml and mannan was used at a concentration of 500 μg/ml, 20 min before adding the bacteria. The concentrations used in the experiments were adapted from our previous studies. Neither the survival of bacteria nor the cells were affected by the reagents added (45).

### Cell Adherence Assay of Fresh Plaque Tissue

Fresh plaque tissues were derived from human coronary arteries of cardiovascular surgery patients recruited at Tongji Hospital for cardiovascular surgery. The plaque tissues were carefully separated from the coronary arteries using anatomical scissor and a scalpel and cut into 2 mm^3^ pieces. The tissue pieces were suspended in RPMI-1640 with 2% FCS and added to a 24-well plate containing 1ml/well. Triplicated wells were established for each experimental group. The tissue samples were added to each well in a sufficient quantity in order to minimize the intergroup variation. 10^6^ bacteria in total at the log phage of the growth is used in the adherence assay. A 50 μl aliquot of the bacterial suspension (OD_600_ = 0.2) was added to each well and incubated at 37°C for 2 h. After 2 h, the plate was carefully washed with PBS. The plaque tissues in the plate were collected and homogenized in 1% Triton X-100 with a tissue grinder. The levels of adherence of bacteria on the plaque sample were calculated by determining the CFU recovered from the lysed tissue samples.

In the inhibition experiment, anti-hDC-SIGN antibody was used at a concentration of 5 μg/ml, mannan at a concentration of 500 μg/ml and core LPS at the concentration of 200 μg/ml, 20 min before the addition of bacteria. The concentrations used in the experiments were adapted from our previous publications (45).

### Plaque Bacteria DNA Profiling

Sequencing of the 16S ribosomal RNA (rRNA) genes is a common method to detect the bacterial composition in different samples (64, 65). 16S rRNA genes have highly conserved sequences interspersed with nine viable regions. Bacterial DNA in AP samples was extracted according to the method described by Ott SJ and colleagues (66). Polymerase chain reaction (PCR) amplification of 16S rRNA genes from the nucleic acids of extracted bacteria was done using the StepOnePlus™ Real-Time PCR System (Thermo Fisher Scientific, Waltham, MA, USA). Precise bacterial profiles were obtained by comparative analyses of the 16S rRNA gene of the DNA extracted from AP samples. Analyses were achieved through single-molecule real-time sequencing (SMRT) technology (Pacific Biosciences, Menlo Park, CA, USA) (38).

Long-read sequencing using SMRT technology can capture the full-length the 16S rRNA sequence and determine the microbial communities of the sample at genus and species levels (67). SMRT can permit achievement of a relatively unbiased view of the bacterial composition of AP samples.

### Immunohistochemistry of Atherosclerotic Plaques

Expression of human DC-SIGN/human langerin was examined using anti-DC-SIGN/anti-langerin antibody-stained cells in all APs. APs were fixed in 4% paraformaldehyde. Staining (hematoxylin and eosin) was employed to demonstrate infiltration of inflammatory cells and the histopathology of AP samples (13). Immunohistochemical analyses for human DC-SIGN/human langerin were undertaken for AP samples and AP samples from asymptomatic patients with cardiovascular disease using anti-human DC-SIGN/human langerin antibodies.

Briefly, AP tissues were cut into pieces of size 2–3 mm and made into paraffin sections. Citrate buffer (pH 6.0) was used for antigen retrieval for 7 min at 100 °C. Afterwards, the slides were blocked with serum for 1 h and incubated with primary antibodies against human DC-SIGN (Pharmingen, San Diego, CA, USA) for 1 h. After washing slides thrice with PBS, they were incubated with the corresponding secondary antibody for 1 h. The slides were examined under an ECLIPSE CI microscope (ECLIPSE CI, Nikon, Japan) and analyzed with ImageJ 1.50 (National Institutes of Health, Bethesda, Maryland, USA) software.

### Extraction of *Proteus mirabilis* Lipopolysaccharide

*P. mirabilis* and *P. mirabilis*-pAY100.1 were analyzed for the O-antigen profile by silver staining. *Y. enterocolitica* cultured at 26°C and 37°C was used as a control in this experiment. Bacterial LPS were extracted with a LPS extraction kit (iNtRON Biotechnology, Daejeon, Korea) according to the protocol sheet from the manufacturer’s instructions. Briefly, the bacterial strains were grown in LB medium at 37°C for 24 h. The bacteria underwent synchronization to an optical density of 1.0 at 600 nm. The bacterial pellets were harvested at room temperature after centrifugation at 13,000 rpm for 30 s. The pellets were washed by three times in phosphate-buffered saline (PBS), suspended in 1 ml bacterial Lysis Buffer and vortexed vigorously to liberate the nucleic acids, proteins, and cell wall components. A 200 μl aliquot of chloroform was added to the suspension. The mixture was mixed vigorously by votexing for 10–20 s and incubated at room temperature for 5 min. The samples were centrifuged at 13,000 rpm for 10 min at 4°C. The LPS in the aqueous phase was collected. An 800 µl aliquot of Purification Buffer was added to the supernatant, mixed and incubated for 10 min at −20°C. The mixture was centrifuged three times for the third times to pellet the extracted LPS (15 min at 13,000 rpm at 4°C). A 1 ml aliquot of 70% EtOH was added to wash the extracted LPS, and the pellet was dried completely. A 70 μl aliquot of Tris-HCL (10 mM pH8.0) was added to the extracted LPS to dissolve the LPS.

### Sodium Dodecyl Sulfate-Polyacrylamide Gel Electrophoresis of *Lipopolysaccharide* Samples From Proteus mirabilis Using Silver Staining

To undertake further analyses of O-antigen expression of *P. mirabilis*, LPS extracted from *P. mirabilis* and *P. mirabilis*-pAY100.1 was analyzed for O-antigen expression by a silver staining kit (Solarbio Science & Technology, Beijing, China) according to a method we have described previously (68).

Purified LPS was resuspended in 5× loading buffer. Samples (10 µl) were subjected to SDS-PAGE. After SDS-PAGE, the gel was transferred to a clean glass dish and fixed with Fixation Solution for 30 min. Next, the gel was transferred to Sensitizing Solution, allowed to incubate for 30 min at room temperature and agitated gently. The gel was washed twice with deionized (10 min each time). After washing, the gel was transferred to Silver Staining Solution, and agitated for 40 min at room temperature. Next, the gel was transferred to Color-substrate Solution and allowed to incubate for 10 min; the reaction was terminated at a set time and Stop Solution was added. Finally, the signals on the gel were acquired by a gel imager (Bio-Rad Laboratories, Hercules, CA, USA).

### Plasminogen Activation Assay

Plasminogen activation was measured, according to a method published previously (51, 59). Briefly, *P. mirabilis* suspended in PBS was mixed with 4 μg of human Glu-plasminogen and 0.45 mM S-2251 in 96-well plates in a total volume of 200 μl at 37°C. The absorption values at 405 nm were measured at 30 min intervals. *Y. pestis* 1418, which possesses high plasminogen activity, was used as the positive control. The results are presented as the difference between each measurement value and the starting value. The *P. mirabilis* used in this study was from a clinical isolate, and our silver staining experiment showed that the LPS lacked the expression of the O-antigen, which indicated that the core LOS of the *P. mirabilis* was exposed. The expression of O-antigen on the rough strain of *P. mirabilis* was reported by other researchers (69). The O-antigen expression might be modified by the *in vivo* environment or antibiotic therapeutic. The rough of Gram negative bacteria shows proteolytic enzyme activity that degrades plasminogen to plasmin (59, 70). The proteolytic enzyme requires rough LPS to function but is specifically inhibited by the O-antigen present in smooth LPS (59, 71). The proteolytic enzyme activity of the bacteria is used as an index to assess the degree of exposure of the LPS core in certain Gram negative bacteria (59, 71). We examined plasmin activity of *P. mirabilis* and further confirmed that *P. mirabilis* used in the current study was a strain that may expose the core LPS.

### Purification of the Core Oligosaccharide of *Proteus mirabilis*

The core oligosaccharide (LPS) of *P. mirabilis* was purified according to a method described previously (45, 72). Purified LPS was hydrolyzed in 0.1 M sodium acetate (pH 4.5) for 4 h at 100°C. A Dowex^®^ 50WX8 column (Sciencelab, Dickinson, TX, USA) was used in centrifugation to remove the sodium. Lipid A was removed by an ultrafiltration device based on a molecular mass of 100 kDa. The remaining core oligosaccharide was lyophilized.

### Curves of Bacterial Growth

*P. mirabilis* and *P. mirabilis*-pAY100.1 were cultured in lysogeny broth (LB) medium with shaking at 37°C until the logarithmic phase was reached. The bacterial suspensions were diluted (1:100) into 5 ml of LB medium and grown at 37°C with shaking at 200 rpm. Bacterial absorption at 600 nm was monitored at 1 h intervals using a spectrophotometer (Thermo-Scientific, Waltham, MA, USA). The growth curve was analyzed with Prism 6 (GraphPad, San Diego, CA, USA).

### Flow Cytometry

We wished to supplement the phagocytosis assay, so flow cytometry was employed. *P. mirabilis* in suspensions were stained by adding carboxyfluorescein diacetate-succinimidyl ester (CFDA-SE) fluorescent probes (Beyotime Biotechnology, Beijing, China) as described previously (48). The bacterial suspension (2 × 10^5^ CFU) was stained with CFDA-SE fluorescent probes (10 μM) for 40 min at room temperature in the dark. The bacterial suspension was washed twice in phosphate-buffered saline (PBS). Cells (1 × 10^5^) in each well were inoculated with bacteria for 2 h in a 24-well plate. Peritoneal macrophages from mice incubated with unlabeled bacteria were served as negative controls. Cells were washed twice with RPMI buffer to remove excess dye. After incubation, the extracellular fluorescent signals were quenched by 0.4% Trypan blue for 10 min. (Trypan blue can quench extracellular fluorescence signals but cannot enter living cells.) Cells were washed twice with PBS and digested by 0.25% Trypsin. Samples were analyzed by flow cytometry at fluorescent intensity of the FL-1 channel.

### Bacterial Infection in an Atherosclerosis Model With Apolipoprotein E Knockout Mice

*P. mirabilis* was cultured at 37°C until reaching absorbance (at 600 nm) of 1. Bacterial suspensions were diluted with PBS at a final concentration of 4×10^7^. After intraperitoneal injection of chloral hydrate, ApoE^−^/^−^ mice and C57BL/6J mice (eight mice in each group) were infected with *P. mirabilis* (100 µl, i.v.). C57BL/6J mice were fed a normal diet for the same period as that used for non-atherosclerotic controls. At 2, 12, 24, and 48 h after injection, mice were anesthetized (chloral hydrate, i.p.). The pleural cavity was opened. Cardiac perfusion was undertaken using 50 ml of physiologic (0.9%) saline through the left ventricle of the mice after making a small incision in the right auricle. After cardiac perfusion, mice hearts were collected and homogenized in 1% Triton X-100 using a tissue grinder. Cardiac levels of bacteria were calculated by determining the CFUs recovered from lysed tissue samples.

### Adherence of Proteus mirabilis to the Heart Tissues of Mice

*P. mirabilis* and *P. mirabilis-*pAY100.1 were used to ascertain the adherence of *P. mirabilis* to the heart tissue of ApoE^−^ **/** ^−^ mice and wild-type mice. The hearts of ApoE^−^ **/** ^−^ mice were cut into pieces at a final area of 1 mm^3^. These tissue pieces were plated in a 24-well plate. Bacteria (2×10^6^) at the log phase of growth were used in the adherence assay. *P. mirabilis* and *P. mirabilis-*pAY100.1 (absorbance at 600 nm = 0.2) were added to each well at a volume of 100 µl and incubated for 2 h at 37°C. Then, the tissue pieces were washed thrice with PBS. Heart tissues were collected and homogenized in 1% Triton X-100 by a tissue grinder. The levels of adherence of bacteria on heart tissues were quantified by determining the CFU recovered from lysed tissue samples.

### Statistical Analyses

All statistical analyses were completed using Prism software, version 6 (Graph Pad, SanDiego, CA, USA). Statistical significance was assessed using Student’s unpaired *t*-test for the univariate analysis of two sets of data.

## Results

### Proteus mirabilis Used in the Study May Not Have Expressed O-Antigen

**Figure 2:**
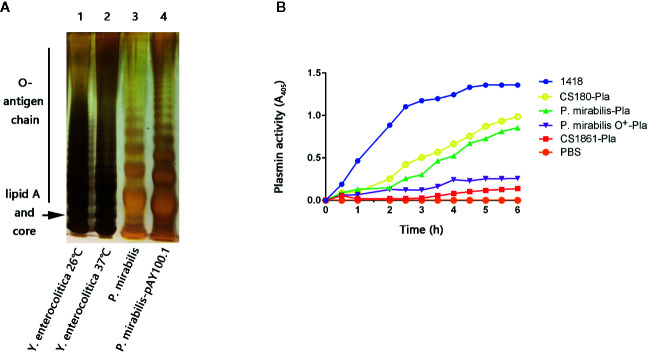
The *P. mirabilis* used in this study is a rough strain. **(A)** Bacterial LPS structures were determined by a silver-stained SDS-PAGE. Lane 1: *Y. enterocolitica* 26°C; lane 2: *Y. enterocolitica* 37°C; lane 3: *P. mirabilis*; lane 4: *P. mirabilis-*pAY100.1. The lipid A and core oligosaccharide, as well as the O-antigen chain are indicated. **(B)** Plasminogen enzyme activity was used to evaluate the level of LPS core exposure of the *P. mirabilis*. The samples presented are: *Yersinia pestis* 1418, *P. mirabilis*-Pla, *P. mirabilis* O^+^-Pla, 180-Pla, 1861-Pla, and PBS. The data were pooled from three independent experiments.

*P. mirabilis* may be associated with AP formation (38, 39). Thus, *P. mirabilis* was chosen to investigate the possible roles of bacterial infection in atherosclerosis. *P. mirabilis* is a Gram-negative bacterium. It can are classified as “smooth” or “rough” based on the presence or absence of O-antigen, respectively (46, 73, 74). It was necessary to determine whether this *P. mirabilis* (a clinical isolate from Wuhan) was a smooth or rough strain. In addition, according to our previous works, O-antigen expression reduces the ability of several species of Gram-negative bacteria to target DC-SIGN, langerin, and DEC-205 (CD205) in humans (43–51). Hence, we also constructed the *P. mirabilis*-pAY100.1 strain, which shows full expression of the O-antigen from *Y. enterocolitica.* SDS-PAGE with silver staining was undertaken on *P. mirabilis* and its isogenic derivative *P. mirabilis*-pAY100.1. Compared with *P. mirabilis*-pAY100.1, *P. mirabilis* did not express the full O-antigen chain (**Figure 2B**). O-antigen expression by *Y. enterocolitica* and *Y. pseudotuberculosis* at 37°C was inhibited (75, 76) (**Figure 2A**).

### Proteus mirabilis Used in our Study was the Rough Strain

A method which measures the activity of PLA enzyme from *Y. pestis* was applied to verify that the *P. mirabilis* used in the present study was a rough strain (59, 70). This method was developed originally by Kukkonen et al. They found that PLA activity can be achieved only in O-antigen-deleting bacteria (59). That is, the higher the PLA activity, the shorter is the O-antigen in Gram-negative bacteria. The same method was also utilized in our recent study on *S. typhimurium* (51). The rationale for using this method is detailed in the Discussion section.

Therefore, we constructed strains of *P. mirabilis*-Pla and *P. mirabilis* O^+^-Pla. *Y. pestis* 1418 is the original strain that codes PLA, and was used as the positive control (51). Meanwhile, we used *Escherichia coli* CS180-Pla and *E. coli* CS1861-Pla as controls (51)*. E. coli* CS180 belongs to the *E. coli* K-12 strain, which usually does not express O-antigen. CS1861 is a CS180 strain that shows full expression of O-antigen. We have used this pair of strains in many of our previous studies (43–51). *P. mirabilis*-Pla expressed active PLA but, in contrast, PLA activity on *P. mirabilis* O^+^-Pla was inhibited (**Figure 2B**). This result further indicated that the *P. mirabilis* used in our study was O-antigen-deleted or that the length of O-antigen was reduced, which could allow exposure of the LPS core.

### Proteus mirabilis Invades CD209-Expressing Macrophages Effectively

**Figure 3:**
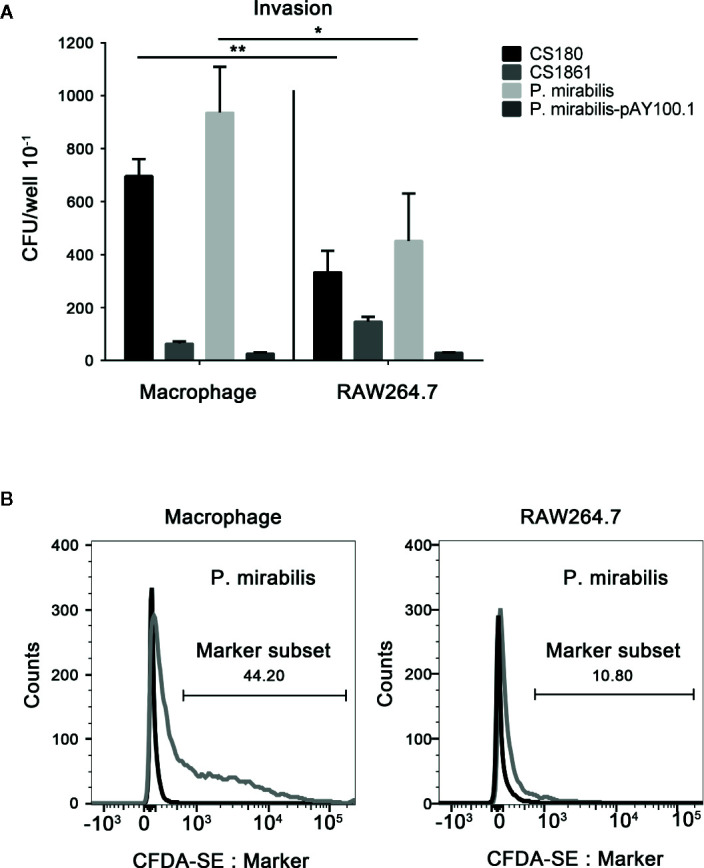
*P. mirabilis* interacts with CD209-expressing macrophages. Gentamicin protection- and flow cytometry-based assays were used to determine the invasion abilities of *Y. pseudotuberculosis* Y1, *E. coli* K-12 (CS180, CS1861), and *P. mirabilis* into primary macrophages that express CD209 and the RAW 264.7 macrophage cell line, which does not express the CD209. Data from the gentamicin protection assay and flow cytometry are shown in **(A, B)**, respectively. Macrophages incubated with labeled and unlabeled bacteria are indicated by gray and black curves **(B)**. The data were pooled from three independent experiments. *P < 0.05, **P < 0.01.

APCs such as macrophages and DCs from the mouse peritoneum express CD209 receptors on their surface, which can interact with the LPS core for invasion (43–46, 49–51). Given the possible exposure of the LPS core for *P. mirabilis* (**Figures 2A, B**), we tested the ability of *P. mirabilis* to invade CD209-expressing macrophages (49–51) and a macrophage cell line that lacks expression of CD209 receptors (77) (RAW264.7) (data not shown). The *E. coli* K-12 strain of CS180 (with rough LPS) and CS1861 (a CS180 strain fully expressing O-antigen) bacteria were used as controls (43). Gentamicin protection and flow cytometry were used to determine the rate of invasion. *P. mirabilis* invaded primary macrophages much more effectively than it invaded RAW264.7 cells (**Figure 3A**). Furthermore, we used CFDA-SE to label *P. mirabilis*. As determined by flow cytometry. 44.2% of macrophages were labeled with high efficiency, whereas 10.8% of the bacteria exhibited weak fluorescence in RAW264.7 cells (**Figure 3B**). The growth rate of *P. mirabilis* was not influenced by expression of exogenous O-antigen (data not shown), as has been shown with other Gram-negative bacteria (48–51).

These results indicated that CD209s might have a role in interacting with *P. mirabilis.*

### Dendritic Cell-Specific Intercellular Adhesion Molecule-3-Grabbing Non-Integrin and Langerin Are Receptors for *Proteus mirabilis*

CD209-expressing macrophages (13, 46, 49–51) phagocytosed more *P. mirabilis* than macrophages that did not express CD209 (**Figure 3A**). Then, we used a stably transfected CHO cell line that expresses hDC-SIGN (CHO-hDC-SIGN) and hLangerin (CHO-hLangerin) to test their ability to phagocytose *P. mirabilis*. We have used these cell lines in many of our previous studies (43–51).

First, the gentamicin-protection assay was used to ascertain the rate of invasion. The *Y. pseudotuberculosis* strain Y1 grown at 26°C has been shown to interact with cells between invasion and its receptor, β-integrin, which is unaffected by other cell-surface molecules (78). Y1 has been used as positive controls. *E. coli* CS180 and CS1861 were used as controls (47, 48, 50, 51).

**Figure 4:**
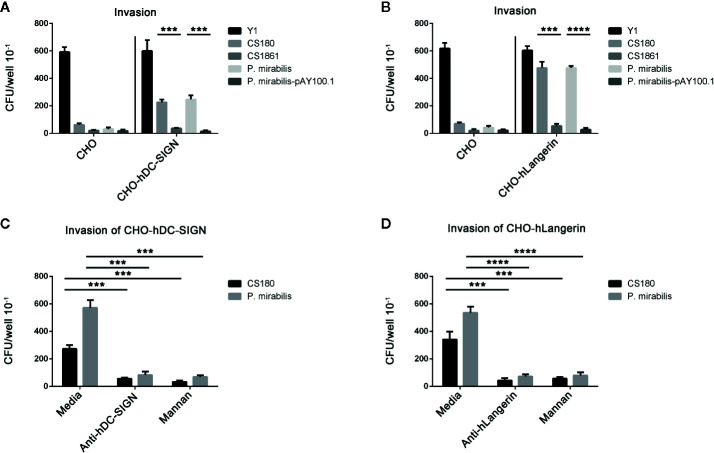
hDC-SIGN and hLangerin are receptors for the rough *P. mirabilis.* ***(a)*** *P. mirabilis* invades the CHO cell line that expresses hDC-SIGN and hLangerin. *Y. pseudotuberculosis* (Y1); *E coli* K-12 (CS180 and CS1861); *P. mirabilis* and *P. mirabilis-*pAY100.1 were examined for their ability to invade CHO/CHO-hDC-SIGN **(A)** and CHO/CHO-hLangerin cells **(B)** using the gentamicin protection assay. The number of phagocytosed bacteria was determined by counting the recovered CFUs. The data were pooled from three independent experiments. ***P < 0.001, ****P < 0.0001. *b* The interactions between *P. mirabilis* with CHO-hDC-SIGN and CHO-hLangerin were blocked by the anti-hDC-SIGN antibody, by mannan oligosaccharides by shielding the ligand of the LPS core by expressing the O-antigen. **(C)** *P. mirabilis* was incubated with CHO-hDC-SIGN for 2 h in the presence or absence of anti-hDC-SIGN and mannan. The phagocytosis rate of *P. mirabilis* was evaluated by recovering the bacteria from the gentamicin protection assay. *E. coli* K12 CS180 was used as the control strain to show the core LPS-dependent interaction with hDC-SIGN. **(D)** Anti-hLangerin and mannan were examined for their ability to inhibit the interaction between CHO-hLangerin and *P. mirabilis*. The data presented were pooled from three independent experiments. ***P < 0.001, ****P < 0.0001.

*P. mirabilis* invaded CHO-hDC-SIGN and CHO-hLangerin cells more effectively than CHO-Neo cells (**Figures 4A, B**). In addition, *P. mirabilis-*pAY100.1 could not promote the invasion of cells expressing CHO-hDC-SIGN or CHO-hLangerin, indicating that O-antigen expression blocked interactions between *P. mirabilis* and CD209/CD207. Therefore, we concluded that hDC-SIGN and hLangerin function as receptors and contribute to the phagocytosis of *P. mirabilis*.

To confirm the specificity of this interaction, we examined whether the host–pathogen interaction could be inhibited by anti-hLangerin antibody, anti-hDC-SIGN antibody, and mannan (antagonist of mannose receptors) (43–46, 48, 50, 51). *E. coli* CS180, which mediates hDC-SIGN/hLangerin-dependent interactions, was used as the control. The invasion of hDC-SIGN and hLangerin by *P. mirabilis* was blocked by addition of both inhibitors (**Figures 4C, D**).

The results shown above demonstrated that a specific interaction between hDC-SIGN/hLangerin-*P. mirabilis* promoted the invasion of this bacterium.

### Dendritic Cell-Specific Intercellular Adhesion Molecule-3-Grabbing Non-Integrin Was Expressed in All Atherosclerotic Plaques of Coronary Arteries Tested, but Not in Healthy Arteries

**Figure 5:**
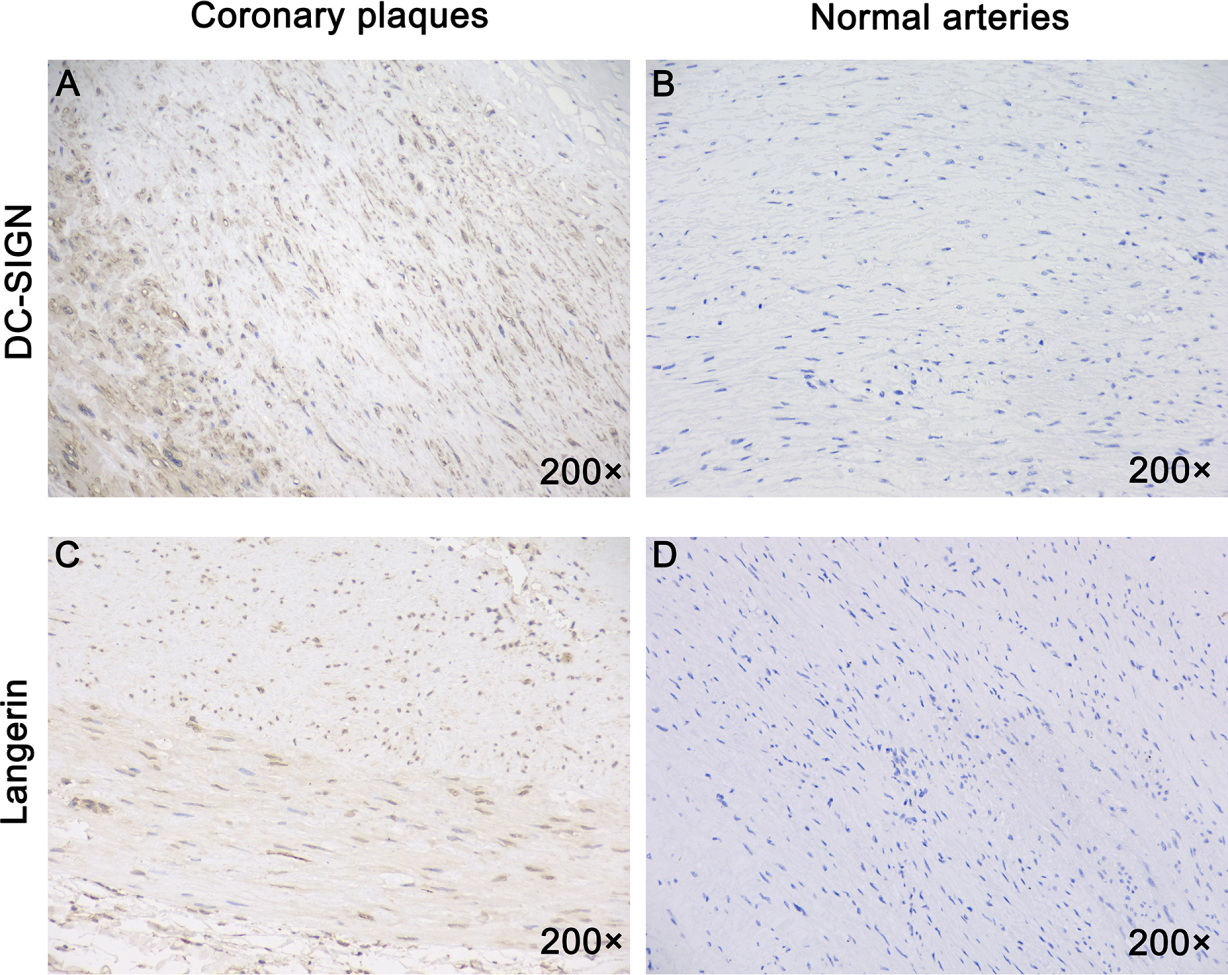
All coronary arteries with plaques, but not healthy human arteries expressed DC-SIGN. Immunostaining for DC-SIGN and Langerin using the brown immunoperoxidase (DAB) technique was described in the materials and methods section. **(A)** DC-SIGN expression was detected in all human coronary plaques. **(B)** DC-SIGN expression was not detected in normal human arteries. **(C)** Langerin was expressed in one human coronary plaque. **(D)** Langerin was not expressed in normal human arteries. Magnification: ×200 in **(A–D.)**

We measured expression of DC-SIGN and langerin in all samples. DC-SIGN expression was detected in the inflamed areas of arteries from all AP samples (**Figure 5A**) but not from the normal arteries (**Figure 5B**). Furthermore, all samples were stained for human langerin. Only one plaque showed langerin expression (**Figure 5C**). Healthy arteries did not express langerin (**Figure 5D**).

In summary, all APs expressed DC-SIGN, and one of them expressed DC-SIGN and langerin.

### Proteus mirabilis Interacted With APs, Which Was Inhibited by Anti-hDC-SIGN, Mannan Oligosaccharides, Core Lipopolysaccharide of *Proteus mirabilis* as Well as Coverage of the Lipopolysaccharide Core With O-Antigen

It was clear that *P. mirabilis* interacted with hDC-SIGN. However, whether *P. mirabilis* would interact with hDC-SIGN detected on APs was not known. Therefore, we tested the ability of *P. mirabilis* to adhere to APs (which were cut into pieces of ~1 mm^3^). The *E. coli* strains of CS180 and CS1861 were used as controls. *P. mirabilis-*pAY100.1 and CS1861 were regarded as “ligand-shielded” strains.

**Figure 6:**
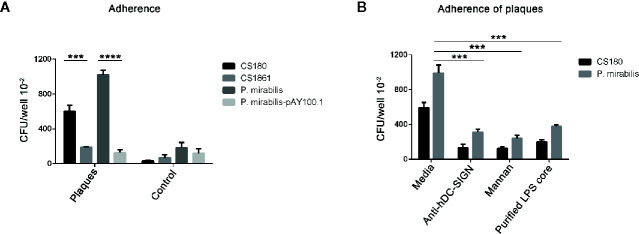
*P. mirabilis* interacts with atherosclerotic plaques, which was inhibited by the anti-hDC-SIGN antibody, mannan oligosaccharide, as well as by covering the ligand. A similar approach to **Figure 4**, the arteries with atherosclerotic plaques were regarded as CHO-DC-SIGN, respectively. **(A)** The two sets of bacteria including *E. coli* K-12 strains (CS180 and CS1861), *P. mirabilis*, and *P. mirabilis-*pAY100.1 were incubated with atherosclerotic plaques to determine the adherence ability of the plaques. **(B)** *P. mirabilis* was incubated with atherosclerotic plaques for 2 h in the presence or absence of anti-hDC-SIGN, mannan, and core oligosaccharide from *P. mirabilis*. *E. coli* K12 CS180 was used as the control strains. The data were pooled from three independent experiments. ***P < 0.001, ****P < 0.0001.

*P. mirabilis* and CS180 adhered to APs more effectively than that observed in healthy arteries and smooth arteries (**Figure 6A**). More importantly, *P. mirabilis-*pAY100.1 barely adhered to APs.

We wished to verify that the interactions of *P. mirabilis* with APs were *via* hDC-SIGN. Hence, we examined if the adherence of *P. mirabilis* to APs could be inhibited by anti-hDC-SIGN and mannan. The adherence of plaques by *P. mirabilis* was reduced by addition of anti-hDC-SIGN and mannan, respectively (**Figure 6B**). Nevertheless, this reduction was not complete, suggesting that additional receptors for *P. mirabilis* were present on APs.

We wished to ascertain if the interaction between *P. mirabilis* and APs was mediated by binding of the core LPS of *P. mirabilis* and CD209. Hence, we further examined the inhibition efficiency of the core LPS from *P. mirabilis* in the interaction with APs. We found that the core LPS purified from *P. mirabilis* could inhibit the binding between bacteria and APs markedly. This result suggested that the O-antigen of *P. mirabilis* was truncated and that the core LPS of *P. mirabilis* was exposed. Hence, we concluded that the interaction between the core LPS of *P. mirabilis* and CD209 cell receptors of the host had major roles in mediating adherence between bacteria and APs.

In summary, these results showed that *P. mirabilis* interacted with APs. This interaction was inhibited by anti-hDC-SIGN, mannan oligosaccharides, core LPS as well as coverage of the LPS core with O-antigen.

### The Proteobacteria Phylum Was One of the Most Dominant Microbiota From One Atherosclerotic Plaque Used in our Study

**Figure 7:**
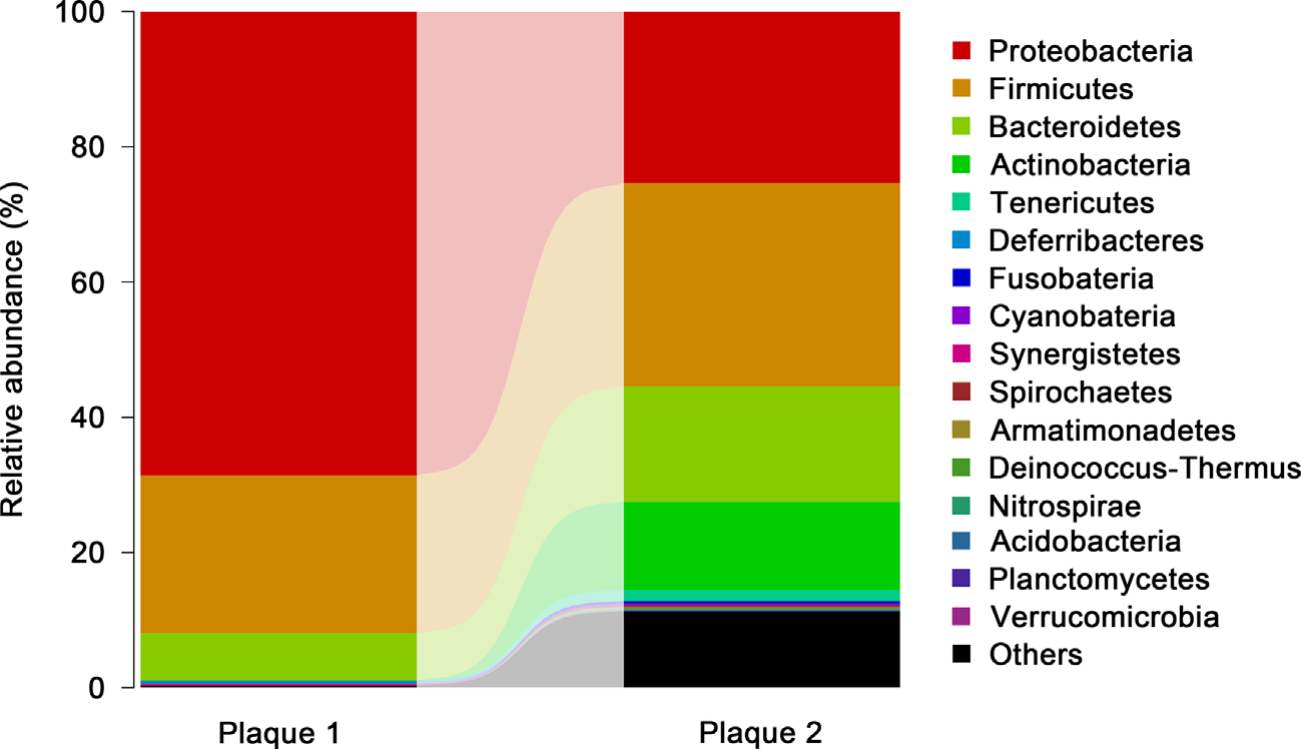
Members of the microbiota that differ in abundance between the plaque samples. The bacterial abundances OTUs of each plaque sample were plotted based on the abundances after sequencing 7,000 selected sequences in each phylum. The proportion rate of OTUs was identified by the length of the column.

We employed standard technology for 16S rRNA sequencing to investigate the microbiota from two AP samples. Across the two samples, 7,000 sequences selected per sample were used for statistical analyses. Operational taxonomic units (OTUs) were applied to illustrate the abundance of each bacterial family at the phylum level in APs. The highest proportion of OTUs identified in AP sample 1 was for bacteria belonging to the phyla Proteobacteria (68.6%), followed by Firmicutes and Bacteroidetes (**Figure 7**). The highest proportion of OTUs in AP sample 2 was for bacteria of phyla Firmicutes(30.1%), followed by Proteobacteria, Bacteroidetes, Actinobacteria, and unclassified phyla.

Collectively, the bacterial profiles from AP sample 1 were similar to data from a study by Bergstrom and coworkers (38). However, the bacterial abundance in AP sample 2 was very different from that in AP sample 1, which revealed the intricacy of the association of bacteria and atherosclerosis development. *P. gingivalis* (the most discussed Gram-negative bacterium due to its involvement in Alzheimer’s disease) belongs to the phylum Bacteroidetes (79–81).

### Proteus mirabilis can Reach the Heart of ApoE−/− Mice

**Figure 8:**
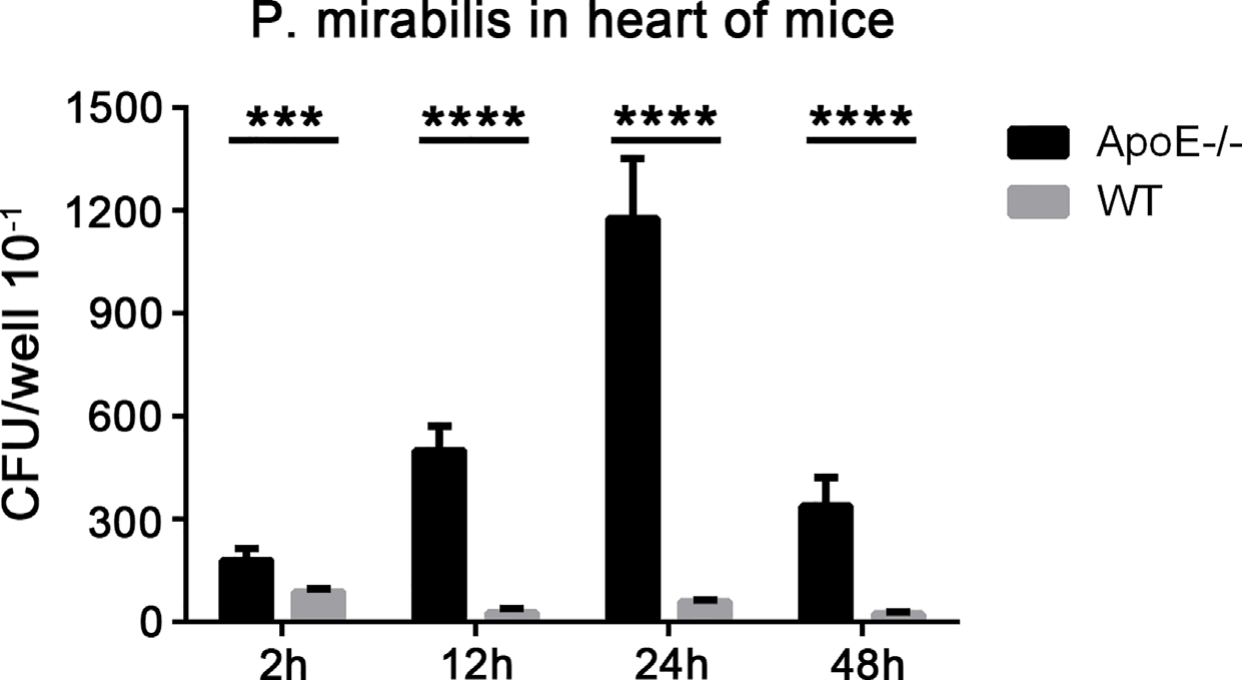
Presence of *P. mirabilis* in mice heart tissues. Mice were infected with *P. mirabilis* (4×10^7^ CFU) through the intravenous route. After 2, 12, 24, and 48 h, the homogenized heart samples were cultured in LB medium, and bacteria were recovered. **(A)** CFU counts in the hearts of ApoE^−^/^−^ mice and wild-type mice. ***P < 0.001, ****P < 0.0001.

We demonstrated that *P. mirabilis* could interact with human coronary APs through DC-SIGN. We wished to further investigate the role of *P. mirabilis* in animals suffering from atherosclerosis. Hence, we infected ApoE^−^/^−^ mice fed a high-fiber diet with *P. mirabilis* through the intravenous route; wild-type mice were used as controls. At 2, 12, 24, and 48 h after infection, substantial levels of bacteria were present in the hearts of ApoE^−^/^−^ mice after grinding mice-heart tissue, and reached a peak at 24 h (**Figure 8**). In contrast, wild-type mice had fewer bacteria in their heart tissue. Significantly, we analyzed the survival curve of mice and found that P. mirabilis was more lethal to the atherosclerotic mice than the wide type mice (data not shown). These results indicated that, as important inflammatory sites, APs can recruit *P. mirabilis* in the blood stream and cause persistent infection.

### Proteus mirabilis Interacts With Heart Tissues From ApoE−/− Mice

**Figure 9:**
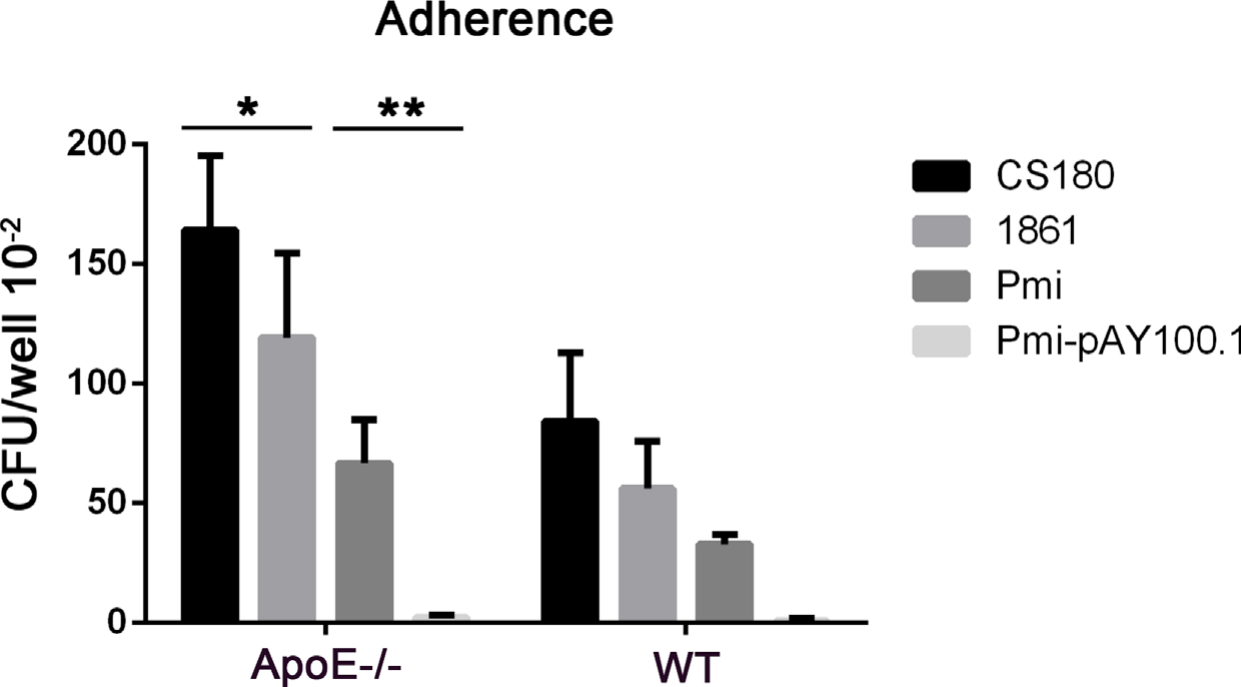
*P. mirabilis* showed higher adherence with the heart tissues of ApoE^−^/^−^ mice compared with that by *P. mirabilis-*pAY100.1. Two sets of bacteria (*Escherichia coli* K-12 strains CS180/CS1861 and *P. mirabilis/P. mirabilis-*pAY100.1) were incubated with the heart tissues of ApoE^−^/^−^ mice to determine bacterial adherence. *P < 0.05, **P < 0.01.

We examined the adherence of *P. mirabilis* with heart tissues from ApoE^−^/^−^ mice and wild-type mice. *P. mirabilis* and *P. mirabilis-*pAY100.1 were incubated with heart tissues from ApoE^−^/^−^ mice and wild-type mice. The *E. coli* K-12 strains of CS180 and CS1861 were used as controls (43). Adherence of *P. mirabilis* with the heart tissues of ApoE^−^/^−^ mice was higher than that in wild-type mice, and *P. mirabilis-*pAY100.1 had lower adherence to the heart tissue of ApoE^−^/^−^ mice compared with that of *P. mirabilis* (**Figure 9**). This result was consistent with the results from the adherence assay of *P. mirabilis* with APs.

## Discussion

At the USA in the early-1900s, there was a notorious case of latent bacterial infection, “Typhoid Mary”, which led to many cases of infections and deaths. As a chef, Mary Mallon was able to transmit a bacterial species, Gram-negative *Salmonella typhi* to many people. This transmission lasted for decades because she was an asymptomatic carrier, who carried a bacterium that could lead to chronic infections in humans (30). Using *S. typhimurium* (the cause of typhoid in mice), we speculated that an interaction between the LPS core and CD209s may contribute to chronic infections by *Salmonella* in the host (51). This speculation was based on a series of studies between the LPS core and C-type lectins that we carried out (43–51).

Besides the lessons learned from Typhoid Mary, studies have unveiled a molecular mechanism of chronic bacterial infections, including Gram-negative bacteria-associated Crohn’s disease, *Helicobacter pylori-*associated chronic atrophic gastritis (ChAG), *P. gingivalis*-associated Alzheimer’s disease and *Mycobacterium tuberculosis-*associated tuberculosis (78, 81–92). These bacteria all share a common characteristic: they can interact with CD209s and survive (and even replicate) within macrophages (34, 78, 84–87). Furthermore, Dr. Helaine and collaborators discovered that phagocytosed *S. typhimurium* can form persistently within macrophages (30). Thus, macrophages, which are supposed to be the “warriors” of innate immunity, are in turn utilized as a “shelter” for a persistent bacterial infection (30, 34).

Increasing evidence suggests that macrophages, inflammation and chronic infection are involved in atherosclerosis pathogenesis (14, 88). We hypothesized that if atherosclerosis is due to chronic infection, it may have similar characteristics to that of chronic infection caused by *Salmonella* species.

We wondered where AP-colonized bacteria were coming from. In general, it is believed that the microbial communities in APs are like those colonizing other parts of the human body; they are highly diverse and have variable features between individuals (89). Koren et al. reported that bacteria from the oral cavity and gastrointestinal tract could be the main sources of bacteria in APs (90). In the present study, we speculated that the consequences of the interaction between *P. mirabilis* and CD209/CD207 might follow a similar molecular mechanism to how the HIV and several Gram-negative bacteria disseminate (47–51, 55). That is, *P. mirabilis* may also hijack APCs by interacting with CD209s for its dissemination to coronary arteries and to promote atherosclerosis development.

We wished to further investigate the role of bacterial infection in atherosclerosis *in vivo* and to elucidate how the interaction between C-type lectins and pathogens can promote the pathogenesis of human atherosclerosis *in vivo*. Hence, we infected *P. mirabilis* through the intravenous route in ApoE^−^/^−^ mice fed a high-fat diet (91–93). Our data showed that *P. mirabilis* could colonize the heart of mice as early as 2 h after injection. Also, the mice showed dyslipidemia, which suggested that atherosclerosis had occurred.

We also utilized the heart tissues of ApoE^−^/^−^ mice and wild-type mice in the bacteria adherence assay. *P. mirabilis* showed higher adherence to the heart tissues of ApoE^−^/^−^ mice compared with that of *P. mirabilis-*pAY100.1 (**Figure 9**). This result further supported the notion that macrophages in APs can bind with *P. mirabilis*, and that O-antigen expression inhibited the binding of bacteria with AP tissues.

In future research, it will be important to ascertain if this mechanism is present in other chronic infectious diseases. For example, colonization of bacteria such as *P. gingivalis* has been noted in Alzheimer’s disease. *P. gingivalis* is involved mainly in gingival and periodontal infections (80, 81). Those studies offered us inspiration that bacterial infection is involved in the development of diseases such as cancer, cardiovascular diseases, and Alzheimer’s disease. Specifically, how these bacteria reached the target organ from peripheral organs attracted our interest.

Previously, we demonstrated consistently that external expression of O-antigen can block exposure of the core LPS (47–51). For example, we showed that a series of Gram-negative bacteria (*S. typhimurium*, *E. coli*, *N. gonorrhoeae*) invaded APCs through binding of the core LPS with DC-SIGN (45). This interaction could be inhibited by external expression of O-antigen (45). We could not exclude the possibility that other factors are involved in this interaction, but the interaction between DC-SIGN and the LPS core plays a major part in the phagocytosis of pathogens. When the ligand or receptor mimics were added, or there was overexpression of the O-antigen, the ability of APCs, macrophages and DCs to phagocytose pathogens was reduced. We have also shown that certain oligosaccharides and polysaccharides can inhibit the interaction between the LPS core and CD209/207 (43–51). In fact, oligosaccharides, glycan and the DC-SIGN-like protein Mermaid can inhibit the binding of DC-SIGN with gp120 of the HIV (45–48, 50, 94–97). Lewis^X^ components (oligosaccharides) from human milk can block the interaction between DC-SIGN and gp120 (98). The structure of Lewis^X^ components is similar to that of core LPS, and purified LPS core can inhibit the interaction between CD209/207 and the LPS core (45, 48, 49). Thus, certain oligosaccharides could be developed as therapeutic candidates for atherosclerosis (48, 99).

## Conclusions

We demonstrated that human DC-SIGN (CD209) expressed on APs could serve as a cellular receptor for *P. mirabilis* (a Gram-negative bacterium that can induce an inflammatory response while colonizing APs) (100). This host–pathogen interaction could be inhibited by blockade of the binding sites for receptors (CD209 or CD207) or carbohydrate ligands (**Figure 6**).

Finally, we would like to borrow a comment from Garcia-Vallejo and van Kooyk: “These manuscripts, call, once again, our attention on the largely unexplored field of bacterial glycosylation, one of the new frontiers of modern glycobiology” (101). The manuscripts they were referring to were four of our publications (45–48). We believe that “these manuscripts” should have included our three recent publications (49–51). These seven publications indicate one fact: blockade of the interactions between the C-type lectins (CD205, CD207, CD209) and carbohydrates of bacteria inhibit bacterial interactions with the host, dissemination, and infection. Blockade of the interaction between human DC-SIGN with bacterial carbohydrates or receptor mimics (e.g., Mermaid) in APs could initiate a new frontier of therapies for atherosclerosis.

## Data Availability Statement

The original contributions presented in the study are included in the article/supplementary materials; further inquiries can be directed to the corresponding authors.

## Ethics Statement

The studies involving human participants were reviewed and approved by the ethical committee of Tongji Hospital, Tongji Medical College, Huazhong University of Science and Technology. The patients/participants provided their written informed consent to participate in this study. The animal study was reviewed and approved by Ethical committee of Tongji Hospital, Tongji Medical College, Huazhong University of Science and Technology.

## Author Contributions

YX and QL performed the experiments, analyzed the data, and wrote the manuscript. CP assisted with the experiments. JK and AA provided critical reagents and advice. ZS provided critical reagents and advice. TC and XW supervised the project, designed the experiments, and modified the manuscript. All authors contributed to the article and approved the submitted version.

## Funding

This work was supported by grants from the National Natural Science Foundation of China [NSFC 81271780 and 81471915 (T.C.)]. CP was supported by grants from the National Research Foundation of Korea (NRF-2014R1A4A1008625, NRF-2017R1D1A1B03028385, and NRF-2019R1F1A 1041700). 

## Conflict of Interest

The authors declare that the research was conducted in the absence of any commercial or financial relationships that could be construed as a potential conflict of interest.
